# Alcohol use disorder among healthcare professional students: a structural equation model describing its effect on depression, anxiety, and risky sexual behavior

**DOI:** 10.1186/s12888-023-04989-1

**Published:** 2023-07-12

**Authors:** Timothy Mwanje Kintu, Mark Mohan Kaggwa, Robinah Namagembe, David Jolly Muganzi, Bernard Raymond Kihumuro, Garvin Ssali Luyinda, Brenda Wafana Nabwana, Muwanguzi Moses, Marvin Nnyombi, Alex Kirega, Jerome Kahuma Kabakyenga, Samuel Maling

**Affiliations:** 1 Faculty of Medicine, Mbarara University of Science and Technology, P.O. Box 1410, Mbarara, Uganda; 2 Department of Psychiatry and Behavioural Neurosciences, McMaster University, Hamilton, ON Canada; 3 College of Health Sciences, Makerere University, P.O. Box 7072, Kampala, Uganda; 4 Faculty of Health Sciences, Busitema University, P.O. Box 1460, Mbale, Uganda; 5 Faculty of Medicine, Gulu University, P.O. Box 166, Gulu, Uganda; 6 Department of Psychiatry, Mbarara University of Science and Technology, P.O. Box 1410, Mbarara, Uganda

**Keywords:** Anxiety, Depression, Risky sexual behavior, Alcohol use disorder, Structural equation modelling, Health professional students, And Uganda

## Abstract

**Background:**

Mental health problems such as depression, anxiety and alcohol use disorders are among the leading causes of disability worldwide. Among university students, alcohol use and poor mental health are associated with risky sexual behavior. Given the syndemic occurrence of these disorders most especially in young adults, we describe the relationship between them so as to guide and intensify current interventions on reducing their burden in this population.

**Methods:**

This was a cross-sectional study based on an online survey among healthcare professional university students that captured sociodemographic characteristics, risky sexual behavior, alcohol use disorder, generalized anxiety disorder, and depression. Structural equation modelling was used to describe the relationship between these variables using RStudio.

**Results:**

We enrolled a total of 351 participants of which 11% (37/351) had Alcohol Use Disorder, 33% (117/351) had depressive symptoms and 32% (111/351) had symptoms of anxiety. A model describing the relationship between these variables was found to fit well both descriptively and statistically [χ^2^ = 44.437, df = 21, p-value = 0.01, CFI = 0.989, TFI = 0.980, RMSEA = 0.056]. All observed variables were found to fit significantly and positively onto their respective latent factors (AUD, anxiety, depression and risky sexual behavior). AUD was found to be significantly associated with risky sexual behavior (β = 0.381, *P <* 0.001), depression (β = 0.152, *P =* 0.004), and anxiety (β = 0.137, *P =* 0.001).

**Conclusion:**

AUD, depression and anxiety are a significant burden in this health professional student population and there’s need to consider screening for anxiety and depression in students reporting with AUD so as to ensure appropriate interventions. A lot of attention and efforts should be focused on the effect of AUD on risky sexual behavior and continued health education is still required even among health professional students.

## Introduction

Alcohol Use Disorder (AUD) is one of the most prevalent psychiatric disorders worldwide and is associated with significant morbidity, mortality and socio-economic burden [1]. In Uganda, 59% of the general population are current drinkers and the prevalence of alcohol use disorders currently stands at approximately 10% [2] which is significantly higher than the African prevalence of 3.7%. Among young adults, alcohol use is an already established burden with the prevalence of heavy episodic drinking being highest among those aged 20–24 years [3].

Globally, university students are known to be heavy drinkers [4] and are reported to consume higher levels of alcohol than their non-university peers [5]. Changes in social contexts, having drinking peers and fewer restrictions due to living away from parents may explain the increase in prevalence, amount and frequency of alcohol use in this age group [6, 7]. Evidence of this problem in Uganda, is drawn from previous studies among students at Makerere University and Mbarara University of Science and Technology (MUST) that found high prevalence rates of maladaptive alcohol use patterns [8, 9]. Studying AUD is very important given its impact on population health and the global burden caused by the harmful use of alcohol. Currently, Africa bears the largest burden of disease and injury attributed to alcohol [3].

Studies have shown that relative to their non-substance using peers, young adults who regularly abuse substances are more likely to be more sexually active at an earlier age [10], have more sexual partners [11] and are more likely to have unprotected sex [12]. A study in Kenya highlighted that 1 in 5 university students engaged in sex after drinking [13]. Harmful use of alcohol has been shown to have a negative impact on HIV infection and transmission by increasing the risk of HIV transmission [14], negatively affecting HIV treatment through alcohol-drug interactions [15] and compromising immune responses thereby leading to increased susceptibility to infection [16].

AUD has also been causally linked to depression and anxiety [17]. Up to 50% of individuals receiving treatment for problematic alcohol use also met diagnostic criteria for one or more anxiety disorders [18]. Studies have previously found that abstainers and heavier drinkers were at higher risk for depression than moderate drinkers [19, 20]. Among university students, two studies established that problem drinking is related to depressive symptoms [8, 20]. Both alcohol use and poor mental health have been identified to be associated with risky sexual behavior [13]. This is because negative thoughts associated with depression [21] affect the way one thinks about themselves and their behaviour which can lead to unhealthy decision-making in several situations including sexual situations [22]. These unsafe sexual behaviours including early sexual debut and having a higher number of sexual partners have previously been established to be more common among the youth due to peer pressure, low self-esteem and perception of low risk associated with these behaviours [23, 24].

Given this previously established relationship between AUD, depression, anxiety and risky sexual behavior, we used structural equation modelling (SEM) to explore the association between these disorders for the first time in this population. SEM is a statistical technique that provides a flexible framework for developing and analyzing complex relationships among multiple variables allowing researchers to test the validity of theory using empirical models [25]. In this study, we explored the magnitude of the effect AUD has on risky sexual behavior, anxiety and depression among study participants. Given the syndemic occurrence of all these disorders and their negative impact most especially in young adults, it was important to study this relationship so as to determine at which point interventions can be implemented for effective reduction in the burden of these disorders in this population.

## Methods

### Study design and setting

This was a cross-sectional study based on an online survey employing quantitative methods during the months of November and December, 2021. The study was carried out among the three largest public health professional universities in Uganda (i.e., Makerere University, Mbarara University of Science and Technology, and Busitema University).

### Study population, sample size, and sampling

The study population included undergraduate health professional students enrolled in the following courses: Bachelor of Medicine and Bachelor of Surgery, Bachelor of Nursing, Bachelor of Physiotherapy, Bachelor of Dental Surgery, Bachelor of Anesthesia and Bachelors of Medical Radiology at the selected universities. Students that did not consent to take part in the study were excluded. The Kish Leslie formula was used to calculate the required sample size [26]. With a maximum variability of 50%, the calculated sample size was 337 participants. In order to reflect the diversity of the population and to prevent oversampling of students from a given university, a stratified random sampling method was used. The number of respondents needed from each university was calculated by multiplying the proportion of health professional students from that university in the overall study population with the overall sample size. Subsequently, the faculty (or college) administrators at each of the universities were contacted for the class lists of each of the selected courses. Each student on the given list was assigned a number. These numbers were picked from each list and fed into a computer, which randomly selected numbers from each list. Students corresponding with the chosen numbers and met the selection criteria to participate in the study were then contacted by their class leader for their emails. The team collected data from 351 participants.

### Data collection procedures

Following ethical approval by the Mbarara University Research and ethics Committee (MUST-2021-166), student leaders in the different courses across the different universities were contacted by a member of the research team and informed about the study procedures, ethical issues and data collection. The nation-wide lockdown during the data collection period necessitated the use of an online tool to collect data and some studies done during the same period had also utilized the same method to collect data [27–30].

The research team and class leaders then shared out a link to Kobo Toolbox, an online survey tool, that hosted the questionnaire to the randomly selected students [31]. The first page of the data collection tool explained the study procedures and objectives before asking a participant to consent. Participants that did not consent were taken to the end of the data collection tool. Participants that consented were linked to the rest of the data collection tool. The questionnaire consisted of demographic and socioeconomic factors of the participants; the AUDIT tool, a Generalized Anxiety Disorder Screen (GAD-7), a screen for depression (PHQ-9) and questions on one’s lifetime sexual behavior.

### Study tools

#### Alcohol Use Disorder Identification Tool

The AUDIT tool was developed by the World Health Organization (WHO) as a method of screening for excessive alcohol consumption in the past 12 months [32]. It detects both AUD (harmful and dependent drinking) and at-risk alcohol consumption (hazardous drinking) which is one of its advantages over other drinking screening tools that mainly focus on harmful and dependent drinking [33]. The AUDIT is a 10-item questionnaire which covers the domains of hazardous alcohol use (questions 1, 2 and 3), dependence symptoms (questions 4, 5 and 6) and harmful alcohol use (questions 7, 8, 9 and 10). A score of 8 or more was indicative of a strong likelihood of hazardous or harmful alcohol consumption [32]. It has previously been validated as a useful tool for screening for alcohol-related problems in university students [34] and was previously used to determine factors associated with alcohol use among students at one university in Uganda [8]. In this study, the Cronbach-alpha for the AUDIT tool was 0.84, hazardous alcohol use was 0.75, dependence symptoms 0.72 and harmful alcohol use was 0.67.

#### Generalized anxiety disorder scale (GAD-7)

The Generalized Anxiety Disorder (GAD-7) questionnaire is a seven-item, self-report anxiety questionnaire designed to assess the patient’s health status during the previous 2 weeks. It was designed by Spitzer and colleagues [35]. The items of the questionnaire inquire about the degree to which the patient has been bothered by feeling nervous, anxious or on edge, not being able to stop or control worrying, worrying too much about different things, having trouble relaxing, being so restless that it is hard to sit still, becoming easily annoyed or irritable and feeling afraid as if something might happen. The scores of this questionnaire are presented from 0 to 21. Scores of 5, 10 and 15 represent cut-off points for mild, moderate and severe anxiety, respectively. Previous studies have established the GAD-7 as a reliable and valid instrument for assessing generalized anxiety in university students [36–38]. In the current study, the Cronbach alpha for the GAD-7 was 0.89.

#### Depression: Patient Health Questionnaire (PHQ-9)

To assess depression, a 9-item depression module from the full Patient Health Questionnaire (PHQ) was used. As a severity measure, the PHQ-9 score can range from 0 to 27, since each of the 9 items can be scored from 0 (not at all) to 3 (nearly every day) [39]. In previous studies, this tool was showed to have good validity and reliability among university students [24, 28, 40, 41] and in resource-constrained settings [42]. In this study, the Cronbach alpha was 0.89.

### Data analysis

All data was analyzed using R version 3.6.0 on R studio Version 1.2.1335. RStudio was used to build the structural equation model in order to test the structural relationship between depression, anxiety and alcohol use disorder as experienced by healthcare professional students in Uganda. The following steps were used to build the model.

Firstly, descriptive statistical analysis (means and standard deviation for normally distributed numerical variables, percentages and frequencies for categorical data) was done to understand the general demographic variables for the participants in the data collected. Secondly, a correlation analysis was conducted to explore the relationship between depression, anxiety and alcohol use disorder among the study participants.

A structural model with four latent variables; alcohol use disorder, depression, anxiety and risky sexual behavior was then defined. AUD was defined by three measurable variables; harmful use, hazardous alcohol use and dependence symptoms, all of which were maintained as continuous variables. Both depression and anxiety were defined by somatic and cognitive-affective symptoms whereas risky sexual behavior was defined by number of sexual partners and change in condom use in the last 12 months.

A number of models were created and compared to identify a model that best fit the data. Path analysis was done to identify relations between the different measured and latent variables (AUD, Depression, anxiety and risky sexual behavior). To test how well the model fit the data, three measures (otherwise known as the absolute fit indices) were used: the chi-squared test, the root mean square error of approximation (RMSEA) and the standardized root mean square residual (SRMR). For the chi-square, a model was considered to fit well if it had an insignificant result at a 0.05 threshold. An upper limit of 0.07 was considered as the cutoff for good fit for the RMSEA whereas a cutoff of less than 0.05 was used for the RMR. For the incremental fit indices (CFI and TLI), a value greater than 0.95 was considered an indicator of good fit [43, 44].

The path coefficient between potential variables was also calculated. Each path coefficient of the model was calculated with its significance being confirmed. A *p* value of less than 0.05 was considered for statistical significance. Modifications were made until a model that meet the defined cutoffs was identified. For this particular population, a model with path coefficients from AUD to depression, anxiety and risky sexual behavior was found to fit best.

## Results

### Participant characteristics

We enrolled 351 participants with a mean age of 25 ± 3.7 years. Of the participants, the majority, 61% (214/351) were male while 39% (137/351) were female. University health professional students from years 1 to 5 were recruited, however year 4 students made up the biggest proportion 171(49%) of the study participants. Twenty-one participants (6%) reported a history of clinically diagnosed mental illness whereas 106 (30%) reported a family history of mental illness. The median number of sexual partners in the last year was 1. Of the study population, 37 participants (11%) had AUD, 117 participants (33%) had depressive symptoms and 111 participants (32%) had symptoms of anxiety (Table 1).

**Table 1:** Characteristics of the study population

Characteristic	Description	Number (percentage)
Age, years (>mean ± *SD*)		24.86 ± 3.68
Gender	Female	137 (39%)
	Male	214 (61%)
University of study	Busitema University	65 (19%)
	Makerere University	173 (49%)
	Mbarara University of Science and Technology	110 (31%)
Year of study	1	10 (3%)
	2	17 (5%)
	3	91 (26%)
	4	171 (49%)
	5	62 (18%)
Marital Status	Single	250 (71%)
	In a relationship	75 (21%)
	Married	26 (7%)
Accommodation	On-campus	135 (39%)
	Off-campus	216 (62%)
Source of tuition	Government funded	155 (44%)
	NGO funded	14 (4%)
	Private funding	182 (52%)
History of mental illness	No	330 (94%)
	Yes	21 (6%)
Family history of mental illness	No	245 (70%)
	Yes	106 (30%)
Childhood environment when growing up	Rural	166 (47%)
	Urban	185 (53%)
Number of sexual partners in last 12 months (median)		1.00 (0.0,2.0)
Alcohol Use Disorder	With AUD	37 (11%)
	No AUD	314 (89%)
Depression	With depression	117 (33%)
	No depression	234 (67%)
Anxiety	With anxiety	111 (32%)
	No anxiety	240 (68%)

### Correlation analysis of the variables

The results of the correlation analysis of the variables are shown in Table 2. The correlation between the depression and anxiety was found to be statistically significant (*r* = 0.754). However, there was no significant or strong correlation found between AUD and depressive symptoms or anxiety symptoms.

**Table 2:** Correlation Analysis of the Variables

Variables	Alcohol Use Disorder	Depression	Anxiety
Alcohol Use Disorder	1		
Depression	0.105	1	
Anxiety	0.102	0.754*	1

*Correlation is significant at the 0.05 level (2-tailed).

### SEM model results

As represented in Table 3, the model was found to fit well descriptively (CFI = 0.989, TFI = 0.980, RMSEA = 0.056) and relatively well statistically (χ^2^ = 44.437, df = 21, p-value = 0.01).

**Table 3:** The Goodness-of-fit Index of Measurement Model

Model	*χ*2	SRMR	CFI	TLI	RMSEA
Statistic values	44.437***	0.045	0.989	0.980	0.056

* ***p*** < 0.05, ** ***p*** < 0.01, *** ***p*** **< 0.001**

**Fig. 1:**
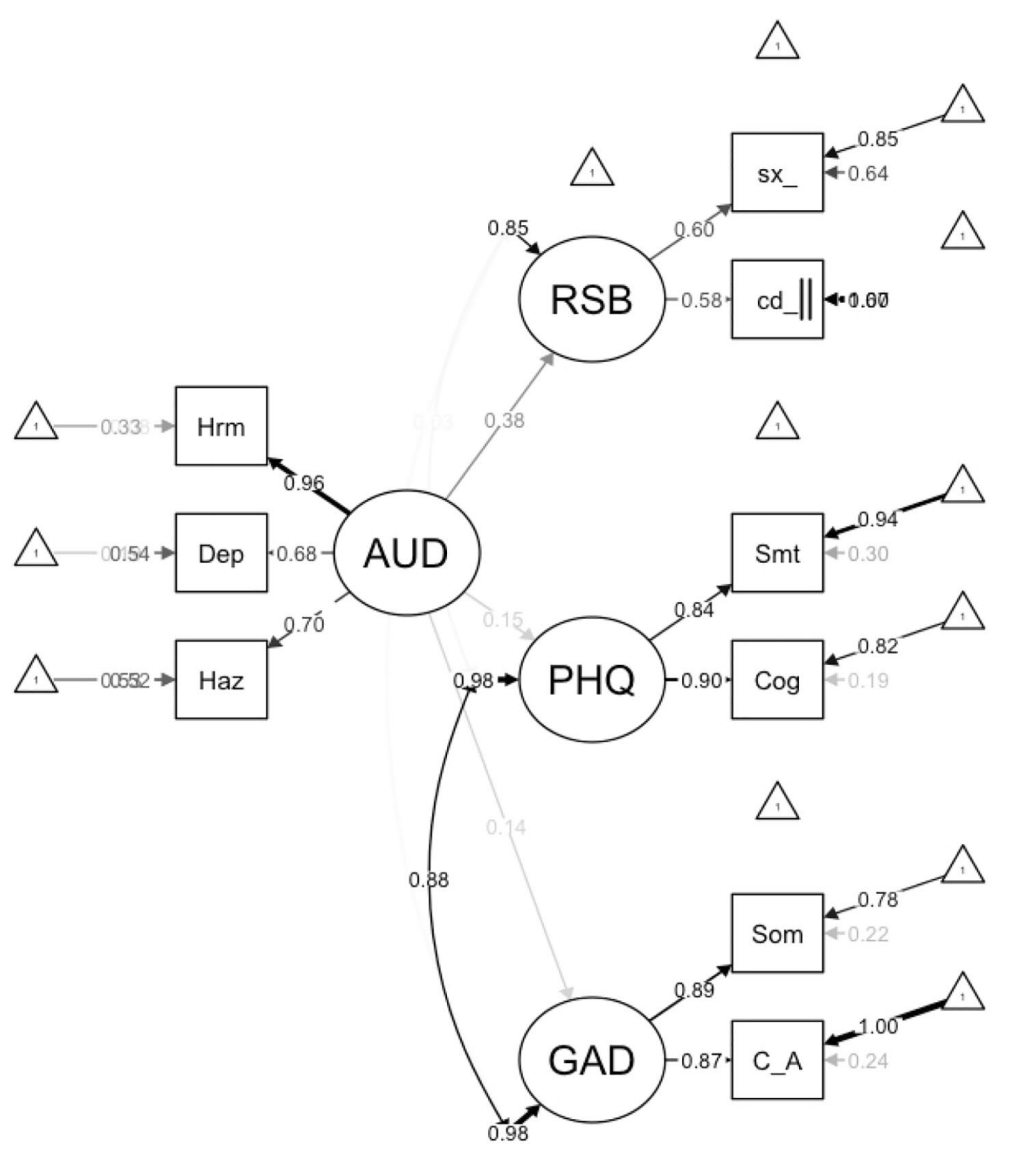
The final model with standardized path coefficients. AUD – Alcohol Use Disorder, Hrm – Harmful use, Dep – Dependence symptoms, Haz-Hazardous use, GAD – Generalized Anxiety Disorder, Som – somatic symptoms, C_A – cognitive-affective symptoms, PHQ – Depression, Smt – somatic symptoms, Cog – cognitive symptoms, RSB – number of sexual partners in last 12 months, cd_ - change in condom use in last 12 months

The final estimated model, with standardized path coefficients, is presented in Fig. 1. In this model, the AUD indicator variables, harmful alcohol use (β = 0.959), dependence symptoms (β = 0.677) and hazardous alcohol use (β = 0.696), loaded significantly onto the AUD factor. Additionally, somatic symptoms (β = 0.839) and cognitive affective symptoms (β = 0.898) loaded significantly onto the depression factor and the two indicator variables, somatic symptoms (β = 0.886) and cognitive-affective symptoms (β = 0.871), loaded on the anxiety factor significantly. The two indicators for risky sexual behavior, condom use over the last year (β = 0.576) and number of sexual partners in the last year (β = 0.599) also loaded significantly.

As indicated in Table 4, AUD was found to be significantly associated with depression (β = 0.152, *P =* 0.004), anxiety (β = 0.137, *P =* 0.001) and risky sexual behavior (β = 0.381, *P <* 0.001). There was also a statistically significant and strong positive inter-factor correlation between depression and anxiety (*r* = 0.876), such that individuals reporting greater anxiety also reported greater depressive symptoms.

**Table 4:** Research model verification results

Path	Standardization factor	Standard error
Risky Sexual Behavior ← AUD	0.381***	0.028
Depression ← AUD	0.15*	0.107
Anxiety ← AUD	0.142**	0.105

* ***p*** < 0.05, ** ***p*** < 0.01, *** ***p*** **< 0.001**

## Discussion

In this study, structural equation modelling was used to determine the relationship between Alcohol Use Disorder (AUD), depression, anxiety, and sexual behavior among health professional students. The AUD factor comprised of self-reported quantitative scores on harmful alcohol use, dependence symptoms and hazardous use of alcohol, all of which are components of Alcohol Use Disorder Identification Tool (AUDIT) developed by the WHO. Each of the mentioned indicator variables loaded significantly onto the single factor, AUD. Subsequently, AUD was found to be a significantly associated with risky sexual behavior, anxiety and depression. Additionally, anxiety and depression in this population of health professional students were found to be highly correlated.

Depressive disorders have been identified as the most common psychiatric disorders among people with AUD [45] and in this study population, AUD was found to be significantly associated with depression. This is similar to findings of a study done among Makerere university students that reported that students who used alcohol were more likely to report depressive symptoms [8]. However, it has also been reported that people with depressive symptoms are more likely to use alcohol to alleviate their symptoms [46] but, in this paper, a structural model with a causal path from AUD to depression was found to fit the data better than a causal path from depression to AUD. This direction of association is similar to findings of Ferguson and colleagues that a causal model in which problems with alcohol use led to increased risk of depression as opposed to depression leading to problems with alcohol use [47].

Previous studies have reported that the presence of either AUD or depression doubles the likelihood of occurrence of the other [48]. To explain this relationship, it has previously been hypothesized that alcohol use disrupts one’s social and economic life, and physical health, these difficulties later predisposing one to depression [48]. Alcohol consumption has also been implicated in the alteration of the brain’s neurotransmitters which may predispose one to depression [48, 49]. Another explanation suggested by McEachin and colleagues is that exposure to alcohol reduces the expression of a key enzyme in folate metabolism reducing the amount of folate [50]. There’s some evidence linking reduced folate levels with increased susceptibility to depression [51]. It has also been argued that both substance use disorders and depression may be caused by similar underlying genetic and environmental factors [48].

The presence of depression in patients with AUD has been noted to affect treatment outcomes because it may affect one’s resolve to quit alcohol [52]. Alternatively, concurrent depression may lead to self-medication with alcohol [29]. Based on the fact that depression may be a direct result of AUD, there’s a chance that depression may remit upon treatment of AUD. It has also been argued that a significant proportion of young adults that present with depressive symptoms may have underlying AUD [53]. This emphasizes the need to screen for alcohol-induced depression in this population of students. A better understanding of this comorbidity may inform intervention and treatment outcomes in this population.

Aforementioned, AUD was also identified as a significant determinant of anxiety symptoms among this population of university students. It has been established that one disorder may be, directly or indirectly, induced by the other. For example, a subject may turn to substance consumption as a means to cope with anxiety. Due to its linkage with alleviation of anxiety symptoms, anxiety and AUD are found to be highly comorbid in the population [54, 55]. This is a more plausible explanation in this population of health care students that are constantly stressed due to the academic pressures of school [56]. However, a study done among university students in UK found conflicting results attaching alcohol use among university students to pleasure rather than a way of dealing with stress or anxiety [57]. Symptoms of anxiety can also be triggered by the stress associated with substance consumption [58]. Owing to the cross-sectional nature of this study, the causal-effect relationship between anxiety symptoms and AUD could not be determined despite some literature emphasizing that anxiety symptoms may predate AUD for this theory to hold. Another theory is based on the biopsychosocial disturbances caused by heavy alcohol consumption which can predispose one to anxiety [55]. Chronic alcohol use has been associated with a GABA deficiency that may induce anxiety [59]. Hazardous drinking has been postulated to lead to anxiety through a noxious combination of greater levels of life stress coupled with relatively poor coping skills.

In this study’s model, the factor risky sexual behavior comprised of number of sexual partners and change in condom use in the last 12 months. Not surprisingly, the relationship between AUD and risky sexual behavior was found to be statistically significant. These findings are consistent with those in a previous study done among MUST students [60] in which alcohol use was found to be associated with having multiple sexual partners. These findings are consistent with those from other studies that report alcohol use and inconsistent condom use among university students [61] and among the youth [62].

Approximately 32% of the participants reported having had more than 1 sexual partner in the previous 12 months and only 15% of participants reported a decrease in their use of condoms in the last 12 months. The relationship between AUD and risky sexual behavior has been postulated to be due to alcohol ability to limit one’s capacity to attend to distal inhibitory cues, thereby biasing attention toward more proximal instigator situational cues such as engaging in unsafe sexual behaviours [63].

The significant relationship found between AUD and depression, anxiety and risk sexual behavior indicates that students need to be educated on the health risks of some of their behaviors. However, there’s also need to evaluate the effect of health education on changing student lifestyles. One limitation of this study is that assessment of AUD was based on self-report and therefore some respondents may have minimized levels of their current drinking. However, a previous study comparing self-report and collateral reporting of one’s use of alcohol indicated self-report of alcohol use had a high validity [64] while another study demonstrated limited benefit of using multiple indicators of alcohol use [65]. Additionally, this was a cross-sectional study and therefore causal relationships between AUD and risky sexual behavior or anxiety and depressive symptoms could not be inferred. Longitudinal studies with a larger sample size are more suitable and recommended to study and understand the causal association between these disorders.

## Conclusion

This study identified alcohol use disorder to be significantly associated with risky sexual behavior, anxiety and depression among health professional students in Uganda. This highlights the need for intensifying current interventions aimed at controlling the levels of alcohol abuse in this population. Going forward, this study highlights the need to routinely do depression and anxiety screens in the young adult presenting with alcohol use problems. Furthermore, there’s need build the capacity of university mental health and addiction recovery programs to respond to and fight against substance use in this risk-population.

## Data Availability

The datasets generated during and/or analyzed during the current study are not publicly available due to ethical reasons but are available from the corresponding author on reasonable request.

## List of abbreviations

AUD: Alcohol Use Disorder
SEM: Structural Equation Modelling
